# Unlocking amyotrophic lateral sclerosis: the role of adiponectin in inflammation and disease progression

**DOI:** 10.3389/fneur.2025.1605822

**Published:** 2025-07-04

**Authors:** Mei Tian, Cheng Xin, Jia Huo, Qi Liu, Hui Dong, Lin Bai, Yafei Wang, Rui Li, Yaling Liu

**Affiliations:** ^1^Key Laboratory of Clinical Neurology, Ministry of Education, Hebei Medical University, Shijiazhuang, China; ^2^Department of Neurology, The Second Hospital of Hebei Medical University, Shijiazhuang, China; ^3^Key Neurological Laboratory of Hebei Province, Shijiazhuang, China

**Keywords:** amyotrophic lateral sclerosis, adiponectin, adiponectin receptor 1, adiponectin receptor 2, inflammatory cytokines, monocytes, macrophages

## Abstract

**Introduction:**

In amyotrophic lateral sclerosis (ALS), immune cells become activated, resulting in a persistent pro-inflammatory milieu and contributing to the development of ALS. Adiponectin produces anti-inflammatory effects via its adiponectin receptor 1 (AdipoR1) and adiponectin receptor 2 (AdipoR2). Currently, there has been limited research conducted on the correlation between adiponectin and inflammation in ALS.

**Methods:**

This cross-sectional study recruited a cohort of 82 ALS patients and 25 controls. Adiponectin and inflammatory mediators in plasma were measured using enzyme-linked immunosorbent assay (ELISA). Furthermore, flow cytometry, immunocytochemistry, and ELISA were employed to examine the levels of AdipoR1, AdipoR2, and inflammatory markers in monocytes and macrophages obtained from ALS patients. The effects of Adiponectin receptor agonists (AdipoRon) on AdipoR expression, inflammatory responses, and macrophages polarization were investigated.

**Results:**

Plasma adiponectin level in ALS patients was markedly lower than controls. This decrease was found to be positively associated with IL-1β, IL-2, IL-6, IL-8, and TNF-α, while negatively correlated with IL-4 and IL-10. Furthermore, there was a positive correlation between plasma adiponectin level and ALS Functional Rating Scale-Revised (ALSFRS-R), and a negative correlation with the disease progression rate (δFS). Mediation research demonstrated that IL-2, or TNF-α, or IL-10 acted as a mediator between adiponectin and δFS. AdipoR1 and AdipoR2 showed a notable increase in expression in peripheral blood monocytes and activated macrophages obtained from ALS patients, concomitant with elevated level of IL-1β. AdipoRon treatment resulted in a decrease in the expression of AdipoR1. Simultaneously, AdipoRon decreased the levels of IL-1β and MHC-II, while boosting the levels of IL-10 and CD206. This regulation enabled the transformation of macrophages from the M1 to the M2 phenotype, therefore aiding in the protection of neurons.

**Conclusion:**

Our findings demonstrated a notable association between adiponectin level and inflammation in the peripheral regions of ALS patients. These results may offer new understanding into the control of inflammation and propose a possible treatment approach for ALS.

## 1 Introduction

Amyotrophic lateral sclerosis (ALS) is a degenerative neurological disorder that causes the gradual loss of both upper and lower motoneurons. It is characterized by a wide range of clinical symptoms and a median survival time of 2–4 years (1). The global crude ALS prevalence was estimated at 4.42 cases per 100,000 population (95% CI 3.92–4.96), with a corresponding incidence rate of 1.59 cases per 100,000 person-years (95% CI 1.39–1.81) (2). The cause and mechanisms of ALS are still not fully understood, and only two medications, riluzole and edaravone, have been approved for use. These treatments have shown some potential effectiveness (3, 4), but their therapeutic outcomes are not adequate.

Neuroinflammation, a key feature of ALS, has attracted growing attention (5). Both individuals with ALS and animal models demonstrate activation of immune cells in the peripheral immune system (PIS) and central immune system (CIS). Notably, although remaining within normal reference ranges, individuals with ALS demonstrated significantly elevated peripheral blood counts of total leukocytes, neutrophils (6), monocytes (7), and natural killer cells (8) compared with healthy controls. It is reported that increased leukocyte and neutrophil counts in the peripheral blood of ALS patients have been linked to rapid disease progression (6). And neutrophil-to-lymphocyte ratio (NLR) has consistently been established as an independent prognostic indicator for survival outcomes in ALS patients (9, 10). Furthermore, in ALS transgenic mice, researchers have found that the presence of activated mast cells, macrophages, and neutrophils in the degenerating motor axons located in sciatic nerves and skeletal muscle (11–13). In the central nervous system (CNS), abundant reactive microglia have been observed in the spinal cord, brain stem, and motor cortex of postmortem tissue from ALS patients (14). In addition, positron emission tomography (PET) imaging of the brains of ALS patients have shown significant activation of microglia (15). As a result, the activation of immune cells causes an increase in the amounts of cytokines and chemokines, including IL-1β, IL-2, IL-6, IL-8, TNF-α, IFN-γ, IL-17, and IL-18, which creates a long-lasting proinflammatory milieu and are strongly linked to the prognosis of the disease (16, 17). Recent data suggests that microglia and other peripheral immune cells, such as lymphocytes and monocytes, are involved in the non-cell autonomous inflammatory process and contribute to the development of ALS (18). Consequently, modulation of neuroimmune dysregulation in ALS has emerged as a pivotal therapeutic frontier. Masitinib, a tyrosine-kinase inhibitor, exhibited therapeutic potential in ALS through its immunomodulatory effects on activated microglia. Specifically, early therapeutic intervention with masitinib significantly mitigated functional and respiratory decline (19, 20). Furthermore, NP001, a small chemical with the ability to control the polarization of monocytes and macrophages, was determined to be safe and well-tolerated. In addition, it showed a tendency to slow down the course of disease (21, 22). Additionally, studies suggested that therapies enhancing regulatory T cells (Tregs) function, such as expanded autologous Tregs with IL-2 and low-dose IL-2 itself, may slow ALS progression safely (23, 24). Hence, the prospective therapeutic approach for ALS is to rectify immunological dysregulation and reduce excessive inflammatory responses.

Adiponectin, a primary adipokine secreted by adipocytes, transmits signals mainly via adiponectin receptor 1 (AdipoR1) and adiponectin receptor 2 (AdipoR2), located on target cells (25). It has attracted significant attention because of its anti-inflammatory qualities. A study investigating multiple sclerosis found that a lack of adiponectin could promote inflammation and damage to CNS (25). In addition, mice lacking adiponectin may experience larger brain infarctions following reperfusion therapy. Providing adiponectin as a supplement can reduce oxidative stress, prevent the activation of NLRP3 inflammasome, and alleviate neurological deficits after cerebral ischemia-reperfusion injury (26, 27). In Alzheimer's disease (AD), a lack of adiponectin was associated with AD-related pathology including phosphorylated Tau, amyloid-β (Aβ) buildup, neuroinflammation and impaired neuronal function (28). Furthermore, several studies have shown that adiponectin or adiponectin receptor agonists (AdipoRon) can regualte the polarization and activity of microglia, reduce the inflammatory responses, and improve the neuropathology in an AD mouse model (29, 30).

Existing research on ALS has demonstrated that patients with ALS had higher level of adiponectin in their peripheral blood compared to healthy individuals (31–33). Bossolasco et al. investigated the sex variations in serum adiponectin levels between ALS patients. However, they did not find any notable disparities in adiponectin level in the blood and cerebrospinal fluid of ALS patients compared to controls (34). Furthermore, Nagel et al. (31) utilized multivariate conditional logistic regression models to show that there was a correlation between adiponectin and the probability of ALS. Besides, Li et al. (32) indicated that adiponectin level correlated positively with ALS functional rating scale-revised (ALSFRS-R), and inversely with disease progression in ALS patients. While adiponectin is linked to ALS pathogenesis, its exact role in peripheral inflammation and macrophage regulation is unclear. There is a lack of systematic research on whether adiponectin directly affects inflammatory mediators in ALS or alters macrophage polarization, despite neuroinflammation's key role in disease progression. We hypothesized that adiponectin deficiency worsened peripheral inflammation in ALS by promoting a pro-inflammatory macrophage phenotype. To investigate, we analyzed the relationship between circulating adiponectin and inflammatory cytokines in ALS patients and assessed AdipoRon's effect on macrophage polarization *in vitro*. This study offered insights into adiponectin's role in neuroinflammation and suggested its potential as a therapeutic target for ALS.

## 2 Materials and methods

### 2.1 Participants

This cross-sectional study recruited all patients diagnosed with ALS from the Department of Neurology at the second hospital of Hebei Medical University, located in Shijiazhuang, China, during February 2023 to April 2024. The revised El Escorial criteria were utilized to diagnose patients with ALS (35). The exclusion criteria included: (1) respiratory insufficiency [defined as Forced vital capacity (FVC) <80% or score <4 on any of the revised ALS functional rating scale (ALSFRS-R) items for dyspnea, orthopnea, or respiratory insufficiency] at enrollment; (2) severe dysarthria and inability to communicate effectively; (3) severe neurological, or psychiatric comorbidities; (4) acute or chronic inflammatory diseases, such as acute pneumonia and rheumatoid arthritis. Age-and sex-matched healthy individuals served as controls. The data collected for ALS patients including the following information: disease duration (time from onset to diagnosis), site of onset (limb/bulbar), disease severity (ALSFRS-R), disease progression rate [δFS: (48-ALSFRS-R at the time of diagnosis)/disease duration]. Besides, body mass index (BMI) was recorded from all recruited participants. We used the STROBE cross sectional checklist when writing our report (36). The study was carried out in compliance with the Declaration of Helsinki and approved by the Ethics Committee of the Second Hospital of Hebei Medical University (protocol code 2024-R285). Prior to the commencement of the investigation, all the participants provided informed consent to participate.

### 2.2 Plasma collection

Blood samples collected in EDTA vacutainers were extracted from 82 ALS patients and 25 controls in the morning after an overnight fast, and then immediately centrifuged for 15 min at ~2,000 × g at room temperature (RT). After centrifugation, the plasma was taken out, divided and frozen at −80°C until further use.

### 2.3 Adiponectin and inflammatory mediators detection

The cryopreserved plasma and cell culture supernatants were utilized for the quantitative detection of adiponectin and inflammatory mediators, including pro-inflammatory cytokines (IL-1β, IL-2, IL-6, IL-8 and TNF-α), anti-inflammatory cytokines (IL-4, IL-10, IL-13, TGF-β), and chemokines (CXCL13, CXCL10, CX3CL1, CCL2), via enzyme-linked immunosorbent assay (ELISA) using commercially available ELISA kits (Bioswamp, Wuhan, China). The optical densities were quantified at a wavelength of 450 nm using an ELISA analyzer (AMR-100, Hangzhou, China). Adiponectin and inflammation mediators concentrations were determined using the guidelines provided by the manufacturer.

### 2.4 Flow cytometry

Peripheral blood monocytes were analyzed using flow cytometry to evaluate the expression levels of AdipoR1, and AdipoR2. The cells in 200 μl of whole blood were stained with antibodies that specifically target cell surface antigens (CD14-APC, 1:100, BD Biosciences, Franklin Lakes, NJ, USA; 555399 and CD16-perCY-CY5.5, 1:100, BD Biosciences, Franklin Lakes, NJ, USA; 560717) for 15 min on ice protecting from light. Then, 2 ml of red blood cell lysis buffer (BD Biosciences, Franklin Lakes, NJ, USA; 349202) was added to completely lyse of red blood cells for 20 min at RT protecting from light. Next, leukocytes were centrifuged at 500 × g for 5 min at 4°C and rinsed twice using phosphate-buffered saline (PBS). Following this, the stained cells were fixed and permeabilized by the addition of 500 μL of fixation and permeabilization solution (BD Biosciences, Franklin Lakes, NJ, USA; 554722) and incubated at RT in the dark for 20 min. The thoroughly resuspended cells were then incubated with anti-AdipoR1 (1:400, Santa Cruz, CA, USA; sc-518030), anti-AdipoR2 antibodies (1:400, Santa Cruz, CA, USA; sc-514045) at 4°C for 30 min protecting from light and the secondary antibody goat anti-mouse IgG (H+L) conjugated with fluorescein-5-isothiocyanate (FITC) (Proteintech, Rosemont, IL, USA; SA00003-1) at 4°C for 20 min in the dark. After the cells were washed with PBS and resuspended in staining buffer, a flow cytometry analysis was performed.

### 2.5 Isolation of peripheral blood mononuclear cells (PBMCs) from blood samples

A commercially available Human peripheral blood lymphocyte separation liquid (Tianjin Haoyang Biological Products Science & Technology Co., Ltd., Tianjin, China; LTS1077) was added to a high-efficiency centrifuge tube (Tianjin Haoyang Biological Products Science & Technology Co., Ltd., Tianjin, China; 601002), and centrifuged at 200 × g for 2 min at RT. Next, the blood samples were added and centrifuged for 30 min at 800 × g. The intermediate mononuclear cell layer was extracted into a new centrifuge tube and centrifuged at 300 × g for 13 min. Following the removal of the supernatant, the pellet was washed multiple times with PBS for subsequent experiments.

### 2.6 *In vitro* culture of macrophages and polarity induction

The previously prepared PBMCs was resuspended in Roswell Park Memorial Institute (RPMI)-1640 medium (Gibco, Waltham, MA, USA; C11875500BT) with 1% penicillin-streptomycin (P/S; Gibco, Waltham, MA, USA; 15140122) and 10% fetal bovine serum (FBS; CellMax, Beijing, China; SA211.02). The culture medium was supplemented with macrophage colony-stimulating factor (M-CSF; PeproTech, East Windsor, NJ, USA; 300-25-10), and the cells were placed in 48-well plates for 7 days. The culture medium was refreshed every 3 days. On the seventh day of cell culture, cells were induced to M1 and M2 phenotype by administering 100 ng/ml lipopolysaccharide (LPS, Sigma-Aldrich, Missouri, USA; L4391) and 20 ng/ml recombinant human IL-4 (IL-4, Peprotech, New Jersey, USA; 200-04) respectively as depicted by Yeudall et al. (37) with minor modification. At the same time, AdipoRon at a concentration of 50 μm was added to investigate the regulatory influence on inflammation.

### 2.7 Immunocytochemistry and confocal microscopy analysis

The medium was removed and fixed with 4% paraformaldehyde in PBS. The cells were then permeated with PBS containing 0.1% Triton-X 100 for 15 min and blocked for 1 h at RT with PBS containing 10% donkey serum. After adding the primary antibody and an overnight incubation, the cells were rinsed three times with PBS and subsequently treated with the corresponding secondary antibody for 1h at RT. The cells were then rinsed and observed using a confocal fluorescence microscope (ZEISS, Oberkochen, Germany; LSM900). The parameters of the microscope were set at the start of each individual imaging process and remained constant throughout. The primary and secondary antibody used in this study were as follows: MHC-II (1:200, Abcam, Waltham, MA, USA; ab23990), CD206 (1:100, R&D Systems, Minnesota, USA; AF2535), IL-1β (1:200, proteintech, Wuhan, China; 16806-1-AP), IL-10 (1:200, Invitrogen, California, USA; 16-7108-81), AdipoR1 (1:400, Santa Cruz, CA, USA; sc-518030), AdipoR2 (1:400, Santa Cruz, CA, USA; sc-514045), donkey anti-mouse IgG H&L 647 (1:1000, Abcam, Waltham, MA, USA; ab150111), donkey anti-rabbit IgG H&L 594 (1:1000, Invitrogen, Shanghai, China; A21207), donkey anti-rat IgG H&L 488 (1:1000, Invitrogen, Shanghai, China; A21208), donkey anti-goat IgG H&L 647 (1:1000, Invitrogen, Shanghai, China; A21447).

### 2.8 Neuron cell lines, cell cultures

NSC34 cells, generated from the fusion of embryonic mouse spinal cord motor neurons and mouse neuroblastoma cells, possess the ability to proliferate while displaying several motor neuron traits (38). In our laboratory, we have generated NSC34 cells that have been transfected with either an empty vector containing green fluorescent protein (GFP; NSC34-E cell) or a plasmid containing the GFP-human SOD1 G93A (hSOD1^G93A^) gene (NSC34-hSOD1^G93A^ cell) (39–41). The cell lines were incubated in Dulbecco's modified Eagle's medium (DMEM; Gibco, Waltham, MA, USA; C11995500BT) containing 10% FBS and 1% P/S. The medium was usually refreshed every 2 days. The cells were passaged at 80%−90% confluence by using a 0.25 % trypsin/EDTA (Gibco, Waltham, MA, USA; 25200056) solution for subsequent experiments.

### 2.9 Cell proliferation and apoptosis detection via live-cell imaging

NSC34-E, and NSC34-hSOD1^G93A^ cells were inoculated into a 96 well plate and allowed to grow overnight, followed by supplementation with the supernatant of macrophages from different groups. Annexin V red dye (Sartorius, Göttingen, Germany; 4641), formerly utilized as an indicator for dead cell, was added to the supernatant in a ratio of 1:200. Real-time cell monitoring was performed using the Incucyte Live-Cell Imaging System (Sartorius, Göttingen, Germany). Phase-contrast images of each well were acquired at 1.5-h intervals over a 72-h period at 10 × magnification. Throughout the imaging process, cells were maintained under standard culture conditions (37°C, 5% CO_2_ humidified atmosphere). The temporal progression of neuronal proliferation and apoptosis was assessed through fluorescence quantification. GFP-positive areas (proliferating cells) and Annexin V-positive regions (apoptotic cells) were digitally segmented using threshold-based image analysis. The cell-value-added rate and cell-value-apoptosis rate can be calculated as follows: (the area of GFP or Annexin V fluorescence at 72 h—the area of GFP or Annexin V fluorescence at 0 h)/the area of GFP or Annexin V fluorescence at 0 h.

### 2.10 Statistical analysis

The Shapiro-Wilk test was employed to assess the normality of the data. Continuous variables that followed normal distributions were presented as mean ± standard deviation, and that non-normal distributions were presented as median with interquartile range. Categorical variables were reported as frequencies. Comparisons between ALS patients and controls were performed using Student's *t*-tests, Manne-Whitney *U* tests. Additionally, Spearman's rank correlation coefficiency test was used for correlation analysis. Furthermore, multivariate linear regression analysis was utilized to adjust for potential confounding factors such as age, sex, BMI, site of onset, and disease duration. In addition, we conducted mediating effect analysis to further investigate the correlation between adiponectin and inflammatory cytokines using Model 4 in the SPSS macro program Process and verified the mediating effects through the bootstrapping procedure. The estimation of direct and indirect effect was performed using a 95% bootstrap confidence interval (CI) derived from a sample of 5,000 bootstrap iterations. If the 95% CI did not include the value of zero, then the effect was deemed to be statistically significant. The statistical analyses were performed using SPSS 22.0 software, and a significance level of *P* < 0.05 was used to determine statistical significance.

## 3 Results

### 3.1 Demographic characteristics, adiponectin and inflammatory mediators levels in both controls and patients with ALS

The analyses comprised a total of 82 patients and 25 control participants. The ALS cohort consisted of 51 males and 31 females, with an average age of 60.53 ± 10.33 years and an average BMI of 23.05 (20.20, 25.55). The control group comprised 14 males and 11 females, with an average age of 57.13 ± 7.16 years and an average BMI of 26.30 (23.54, 27.25) (Table 1). The expression levels of adiponectin and inflammatory mediators in ALS patients and controls were presented separately in Table 1. The level of plasma adiponectin was markedly reduced in ALS patients (*P* < 0.05). The concentrations of pro-inflammatory cytokines, including IL-1β, IL-2, IL-6, IL-8, and TNF-α, were notably higher in the patients with ALS when compared to the controls (*P* < 0.05). Among the anti-inflammatory cytokines, only IL-10 showed a clear distinction between the two groups. The levels of IL-4 [462.66 (380.70, 555.82) vs. 498.45 (352.35, 610.67), *P* = 0.480], IL-13 [224.64 (163.44, 281.77) vs. 229.15 (181.43, 291.04), *P* = 0.524], TGF-β [492.98 (333.23, 590.89) vs. 468.71 (340.43, 609.11), *P* = 0.982] did not show significant difference between ALS patients and controls. Additionally, there were no obvious difference in the concentrations of chemokines CXCL13 [193.43 (151.22, 228.43) vs. 184.19 (134.93, 224.37), *P* = 0.381], CXCL10 [452.52 (376.24, 540.13) vs. 425.73 (374.39, 570.54), *P* = 0.749], CX3CL1 [3.08 (2.42, 3.79) vs. 3.05 (2.57, 3.75), *P* = 0.965], CCL2 [357.08 (294.46, 447.02) vs. 360.52 (318.30, 433.48), *P* = 0.988] between ALS patients and controls.

**Table 1:** Patients with ALS and control subjects were compared on their demographic characteristics, adiponectin levels and levels of inflammatory mediators.

	**ALS (*n* = 82)**	**Controls (*n* = 25)**	***P*-value**
Age (years)	60.53 ± 10.33	57.13 ± 7.16	0.127
Sex ratio (men/women)	51/31	14/11	0.58
BMI	23.05 (20.20, 25.55)	26.30 (23.54, 27.25)	**0.003** ^**^
**Adipokine**			
Adiponectin (ng/ml)	54.08 (39.76, 65.19)	60.3 (54.32, 72.28)	**0.047** ^*^
**Pro-inflammatory cytokines**			
IL-1β (pg/ml)	313.83 (257.07, 357.32)	267.85 (219.18, 328.67)	**0.035** ^*^
IL-2 (pg/ml)	303.42 (250.52, 354.63)	277.14 (221.41, 313.70)	**0.025** ^*^
IL-6 (pg/ml)	120.34 (96.70, 137.48)	100.59 (77.66, 131.91)	**0.047** ^*^
IL-8 (pg/ml)	323.83 (258.80, 398.58)	286.21 (217.27, 359.09)	**0.050** ^*^
TNF-α (pg/ml)	334.55 (280.77, 394.04)	283.76 (212.79, 347.29)	**0.030** ^*^
**Anti-inflammatory cytokines**			
IL-4 (pg/ml)	462.66 (380.70, 555.82)	498.45 (352.35, 610.67)	0.480
IL-10 (pg/ml)	104.82 (80.05, 134.58)	120.64 (93.74, 161.77)	**0.046** ^*^
IL-13 (pg/ml)	224.64 (163.44, 281.77)	229.15 (181.43, 291.04)	0.524
TGF-β (pg/ml)	492.98 (333.23, 590.89)	468.71 (340.43, 609.11)	0.982
**Chemokines**			
CXCL13 (pg/ml)	193.43 (151.22, 228.43)	184.19 (134.93, 224.37)	0.381
CXCL10 (pg/ml)	452.52 (376.24, 540.13)	425.73 (374.39, 570.54)	0.749
CX3CL1 (ng/ml)	3.08 (2.42, 3.79)	3.05 (2.57, 3.75)	0.965
CCL2 (pg/ml)	357.08 (294.46, 447.02)	360.52 (318.30, 433.48)	0.988

Data are mean ± SD or median (interquartile range). ALS, amyotrophic lateral sclerosis; BMI, body mass index; ^*^P < 0.05, ^**^P < 0.01.

### 3.2 A correlation analysis between plasma adiponectin level and the clinical status of patients with ALS

**Figure 1:**
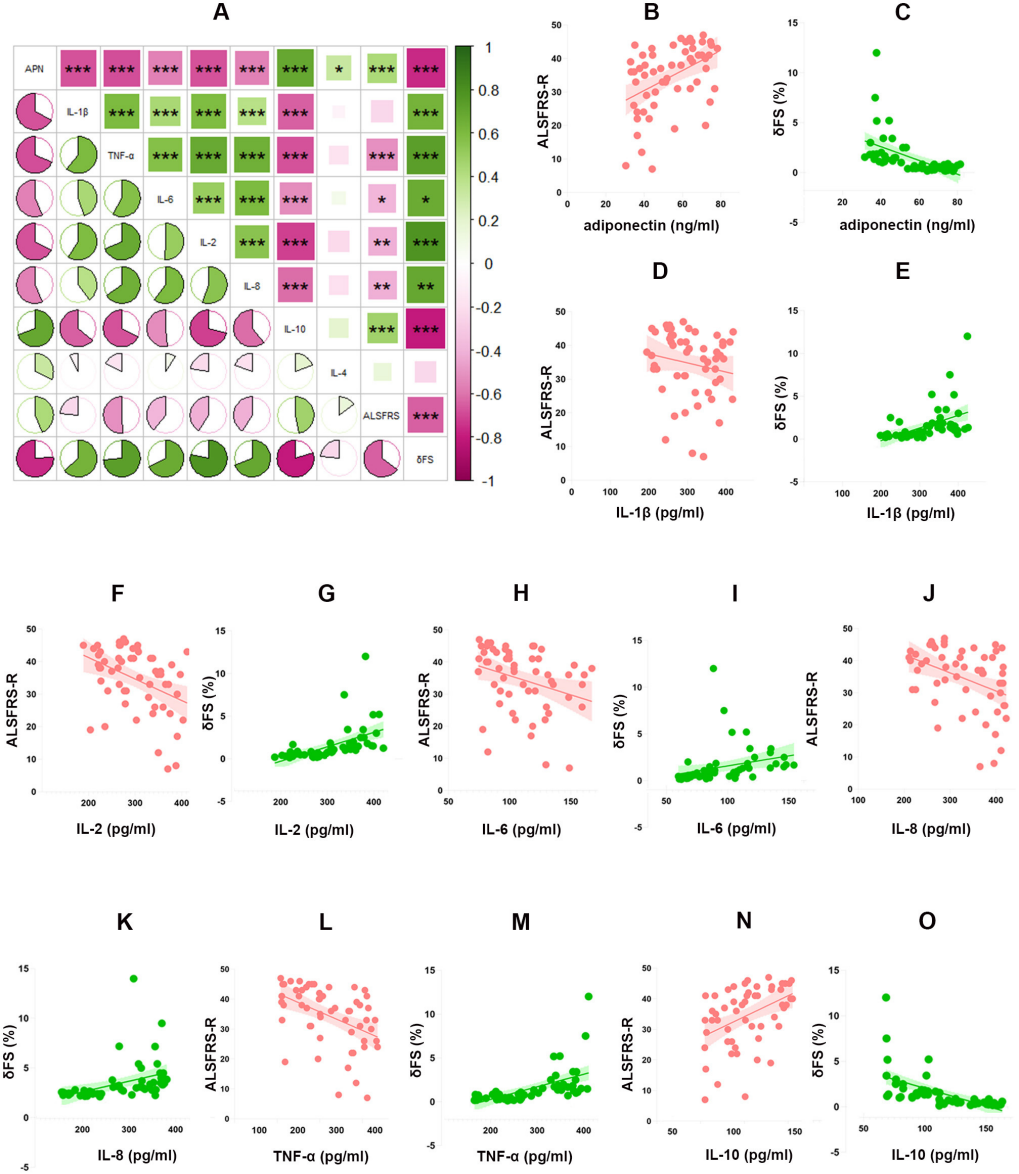
Plasma adiponectin and inflammatory cytokines and their correlations with ALS clinical status. **(A)** The relationship between adiponectin, inflammatory cytokines, and clinical status, as well as the relationship between adiponectin and inflammatory cytokines, was assessed with Spearman analyses. The negative correlations were indicated by pink, and positive correlations were represented by green. The fan-shaped areas indicated the magnitude of the correlation coefficients. *P < 0.05; **P < 0.01; ***P < 0.001. Multivariate analyses on the relationship between adiponectin and ALSFRS-R **(B)**, δFS **(C)**, the relationship between IL-1β and ALSFRS-R **(D)**, δFS **(E)**, the relationship between IL-2 and ALSFRS-R **(F)**, δFS **(G)**, the relationship between IL-6 and ALSFRS-R **(H)**, δFS **(I)**, the relationship between IL-8 and ALSFRS-R **(J)**, δFS **(K)**, the relationship between TNF-α and ALSFRS-R **(L)**, δFS **(M)**, the relationship between IL-10 and ALSFRS-R **(N)**, δFS **(O)**. APN, Adiponectin; ALSFRS-R, revised ALS functional rating scale; δFS = 48-ALSFRS-R at the time of diagnosis/disease duration.

Out of 82 ALS patients, detailed clinical information were collected for 57 individuals. The analysis indicated that plasma adiponectin level was generally lower in male patients compared to female patients, with a mean value of 44.82 (37.65, 63.93) for males and 62.02 (47.87, 71.60) for females (*P* = 0.029) (Table 2). Moreover, among the total of 57 patients, 43 experienced the beginning of symptoms in their limbs, whereas 14 in their bulbar regions. Comparative analysis between the two groups showed no difference in plasma adiponectin level, with a mean value of 62.83 (48.96, 66.03) for limb onset and 48.57 (39.23, 67.47) for bulbar onset (*P* = 0.286) (Table 2). Additionally, we investigated the correlation between adiponectin level and ALSFRS-R, δFS. Spearman correlation analyses indicated a significantly positive correlation between adiponectin level and ALSFRS-R (*r* = 0.434, *P* = 0.001), and an inverse relationship between adiponectin level and δFS (*r* = −0.761, *P* < 0.001) (Figure 1A; Supplementary Table 1). After adjusting for age, sex, BMI, site of onset, and disease duration, adiponectin was independently associated with ALSFRS-R and δFS (*P* < 0.05, Figures 1B, C; Table 3).

**Table 2:** An analysis of plasma levels of adiponectin and inflammatory mediators among ALS patients with different genders and sites of onset.

	**Men (*n* = 35)**	**Women (*n* = 22)**	***P*-value**	**Bulbar (*n* = 14)**	**Limb (*n* = 43)**	***P*-value**
Age (years)	58.82 ± 10.85	54.45 ± 10.94	0.831	57.34 ± 11.25	64.36 ± 7.25	**0.033** ^*^
BMI	24.22 (20.76, 26.12)	22.10 (19.70, 25.96)	0.466	24.05 (20.96, 26.12)	21.05 (18.34, 26.03)	0.295
Disease duration (months)	12 (6,24)	12 (6, 18)	0.889	11 (6, 20)	15 (11, 19.5)	0.104
ALSFRS-R	36 (26, 41)	38 (32.50, 43.25)	0.290	36 (26, 41)	39 (34.75, 45)	0.112
δFS	1.08 (0.46, 1.67)	0.78(0.37, 1.79)	0.471	1.10 (0.47, 1.83)	0.47 (0.27, 0.90)	**0.019** ^*^
**Adipokine**						
Adiponectin (ng/ml)	44.82 (37.65, 63.93)	62.02 (47.87, 71.60)	**0.029** ^*^	48.57 (39.23, 67.47)	62.83 (48.96, 66.03)	0.286
**Pro-inflammatory cytokines**						
IL-1β (pg/ml)	320.44 ± 59.71	288.95 ± 64.95	0.066	314.76 ± 63.73	288.39 ± 59.00	0.177
IL-2 (pg/ml)	307.51 ± 57.97	290.46 ± 63.40	0.302	328.82 (265.87, 365.06)	273.45 (232.05,303.32)	**0.040** ^*^
IL-6 (pg/ml)	111.93 ± 25.78	106.86 ± 25.50	0.470	115.31 (91.75,129.96)	93.71 (80.81,108.14)	**0.040** ^*^
IL-8 (pg/ml)	326.25 (266.79,398.47)	332.51 (277.19,400.85)	0.743	365.21 (287.40,403.02)	260.68 (226.01,318.31)	**0.004** ^**^
TNF-α (pg/ml)	321.44 (281.28,386.34)	271.20 (214.41,387.40)	0.098	343.95 (274.28,404.09)	273.05 (243.35,300.90)	**0.015** ^*^
**Anti-inflammatory cytokines**						
IL-4 (pg/ml)	452.31 ± 106.92	456.23 ± 118.42	0.898	454.47 ± 104.85	451.84 ± 130.55	0.939
IL-10 (pg/ml)	100.00 ± 31.64	107.90 ± 30.52	0.356	91.49 (69.58, 114.76)	142.60 (99.94, 147.93)	**0.001** ^**^
IL-13 (pg/ml)	239.36 (151.90, 304.01)	213.88 (166.34, 255.42)	0.658	239.36 (151.90, 282.07)	217.99 (168.17, 278.49)	0.853
TGF-β (pg/ml)	493.38 (382.67, 588.06)	464.40 (294.60, 583.22)	0.658	498.96 (406.24, 577.57)	371.49 (285.70, 610.11)	0.321
**Chemokines**						
CXCL13 (pg/ml)	200.23 (167.01, 230.62)	157.49 (133.78, 218.00)	**0.040** ^*^	178.78 (140.49, 228.12)	201.36 (177.92, 229.49)	0.250
CXCL10 (pg/ml)	473.17 (378.40, 526.50)	412.01 (347.51, 567.57)	0.507	469.94 ± 101.82	408.41 ± 90.16	**0.049** ^*^
CX3CL1 (ng/ml)	3.05 (2.43, 3.57)	3.48 (2.43, 3.91)	0.350	2.84 (2.41, 3.65)	3.59 (2.88, 3.93)	0.085
CCL2 (pg/ml)	354.62 (296.30, 448.64)	413.12 (315.12, 413.12)	0.502	361.51 (301.18, 448.64)	332.76 (292.03, 332.76)	0.394

Data are mean ± SD or median (interquartile range). BMI, body mass index; ALSFRS-R, revised ALS functional rating scale; δFS= 48-ALSFRS-R at the time of diagnosis/disease duration; ^*^P < 0.05, ^**^P < 0.01.

**Table 3:** Multivariate linear regression analyses of ALSFRS-R and disease progression rate.

	**ALSFRS-R**		δ**FS**	
	***B*** **(95% CI)**	***P*** **-Value**	***B*** **(95% CI)**	***P*** **-Value**
APN (ng/ml)	0.434 (0.271, 0.597)	**<0.001** ^**^	−0.067 (−0.103, −0.031)	**0.001** ^**^
IL-1β (pg/ml)	−0.055 (−0.102, −0.008)	**0.022** ^*^	0.013 (0.003, 0.022)	**0.008** ^**^
IL-2 (pg/ml)	−0.117 (−0.157, −0.076)	**<0.001** ^**^	0.015 (0.005, 0.025)	**0.003** ^**^
IL-6 (pg/ml)	−0.151 (−0.251, −0.051)	**0.004** ^**^	0.011 (−0.011, 0.033)	0.316
IL-8 (pg/ml)	−0.083 (−0.121, −0.046)	**<0.001** ^**^	0.007 (−0.002, 0.016)	0.110
TNF-α (pg/ml)	−0.084 (−0.117, −0.051)	**<0.001** ^**^	0.014 (0.007, 0.021)	**<0.001** ^**^
IL-10 (pg/ml)	0.219 (0.132, 0.326)	**<0.001** ^**^	−0.036 (−0.054, −0.017)	**<0.001** ^**^

Adjusted for age, sex, BMI, site of onset, and disease duration; ALSFRS-R, revised ALS functional rating scale; APN, Adiponectin; CI, confidence interval; δFS= 48-ALSFRS-R at the time of diagnosis/disease duration; ^*^P < 0.05; ^**^P < 0.01.

### 3.3 A correlation analysis between plasma inflammatory mediators levels and the clinical status of patients with ALS

A comparative examination of plasma inflammatory markers in male and female patients revealed that the level of CXCL13 was significantly greater in men compared to women, with a mean value of 200.23 (63.61) for men and 157.49 (84.22) for women (*P* = 0.040) (Table 2). Furthermore, in comparison to limb onset patients, bulbar onset patients presented raised plasma concentrations of IL-2, IL-6, IL-8, TNF-α, and CXCL10, whereas the level of IL-10 was distinctly lower (*P* < 0.05) (Table 2). Spearman correlation analyses indicated that IL-1β (*r* = 0.639, *P* < 0.001), IL-2 (*r* = 0.786, *P* < 0.001), IL-6 (*r* = 0.674, *P* < 0.001), IL-8 (*r* = 0.694, *P* < 0.001) and TNF-α (*r* = 0.738, *P* < 0.001) were positively correlated to δFS, while showing negative correlations with ALSFRS-R except IL-1β (IL-2: *r* = −0.412, *P* = 0.001; IL-6: *r* = −0.399, *P* = 0.002; IL-8: *r* = −0.410, *P* = 0.002; TNF-α: *r* = −0.510, *P* < 0.001, IL-1β: *r* = −0.234, *P* =0.08,). Besides, IL-10 level was positively related to ALSFRS-R (*r* = 0.471, *P* < 0.001) and negatively associated with δFS (*r* = −0.801, *P* < 0.001) (Figure 1A; Supplementary Table 1). Further multivariate analyses revealed independently significant correlations between IL-1β, IL-2, IL-6, IL-8, TNF-α, IL-10, and ALSFRS-R (*P* < 0.05). And IL-1β, IL-2, TNF-α, and IL-10 were independently related to δFS (*P* < 0.05, Figures 1D–O; Table 3). However, there was no correlation observed between other inflammatory mediators, including IL-4, IL-13, TGF-β, CXCL13, CXCL10, CX3CL1, CCL2 and ALSFRS-R, δFS in ALS patients (Supplementary Table 1).

### 3.4 A correlation analysis between plasma adiponectin level and inflammatory mediators levels of patients with ALS

Spearman correlation analyses revealed significantly inverse correlations of plasma adiponectin level with IL-1β (*r* = −0.674, *P* < 0.001), IL-2 (r = −0.676, *P* < 0.001), IL-6 (*r* = −0.564, *P* < 0.001), IL-8 (*r* = −0.566, *P* < 0.001), TNF-α (*r* = −0.682, *P* < 0.001), and positive correlations with IL-4 (*r* = 0.320, *P* = 0.015) and IL-10 (*r* = 0.693, *P* < 0.001) in the cohort of 57 ALS patients (Figure 1A). Nevertheless, no correlation was examined between adiponectin level and other inflammatory mediators levels (IL-13: *r* = −0.071, *P* = 0.602; TGF-β: *r* = −0.143, *P* =0.289; CXCL13: *r* = −0.136, *P* = 0.311; CXCL10: *r* = −0.162, *P* = 0.228; CX3CL1: *r* = 0.073, *P* = 0.588; CCL2: *r* = 0.022, *P* = 0.873; Supplementary Table 2).

### 3.5 An analysis of the mediation effect of adiponectin on disease progression rate in patients with ALS using inflammatory cytokines

ALS patients' clinical status was affected by plasma adiponectin as well as plasma IL-1β, IL-2, IL-6, IL-8, TNF-α, and IL-10, and plasma adiponectin significantly correlated with these inflammatory cytokines. Thus, further study was conducted to determine if IL-1β, IL-2, IL-6, IL-8, TNF-α, and IL-10 mediated the relationship between adiponectin and ALSFRS-R, δFS by using a simple mediation analysis model. The results showed that adiponectin did not have a direct effect on δFS when IL-2, or TNF-α, or IL-10 was used as mediating variables, but it had significant indirect and total effect on δFS when these variables were used respectively (*P* < 0.05). This implied that the impact of adiponectin on δFS was solely mediated by IL-2, or TNF-α, or IL-10. However, analyses of adiponectin with ALSFRS-R revealed no significant mediation effects via IL-1β, or IL-2, or IL-6, or IL-8, or TNF-α, or IL-10 (Table 4).

**Table 4:** An analysis of the mediation effect of adiponectin via inflammatory cytokines on disease progression rate.

	**Total effect**	**Direct effect**	**Mediation effect**
Mediator	Effect size (95% CI)	Effect size (95% CI)	Effect size (95% CI)
IL-2	−0.072 (−0.103, −0.041)	−0.041 (−0.084, 0.001)	−0.031 (−0.056, −0.011)
TNF-α	−0.072 (−0.103, −0.041)	−0.034 (−0.077, 0.009)	−0.038 (-0.081, −0.013)
IL-10	−0.072 (−0.103, −0.041)	−0.033 (−0.075, 0.009)	−0.040 (-0.074, −0.015)

CI, confidence interval; Total effect: the effect of adiponectin on disease progression rate; Direct effect: the effect of adiponectin on disease progression rate that is not explained by the mediator; Mediation effect: the effect of adiponectin on disease progression rate acting through the mediator; If the 95% CI did not contain zero, the effect was statistically significant.

### 3.6 Evaluation of AdipoRs levels in monocytes and monocyte-derived macrophages isolated from the blood of patients with ALS

Based on the known connection between adiponectin level and peripheral inflammatory cytokines, we hypothesized that adiponectin could have a regulatory function in inflammation among ALS patients. Mounting evidence indicated that peripheral blood monocytes and macrophages derived from ALS patients displayed heightened expression of proinflammatory genes (42). Besides, accumulating data indicated that invading monocyte-derived macrophages were functionally equivalent to microglia (43), and that modified macrophages in the peripheral regions could modify and influence the reactivity of microglia and extend ALS survival (44). Consequently, our work primarily focused on investigating the role of adiponectin in regulating inflammation in peripheral monocytes and macrophages in ALS patients, taking advantage of the convenience of acquiring peripheral blood samples.

**Figure 2:**
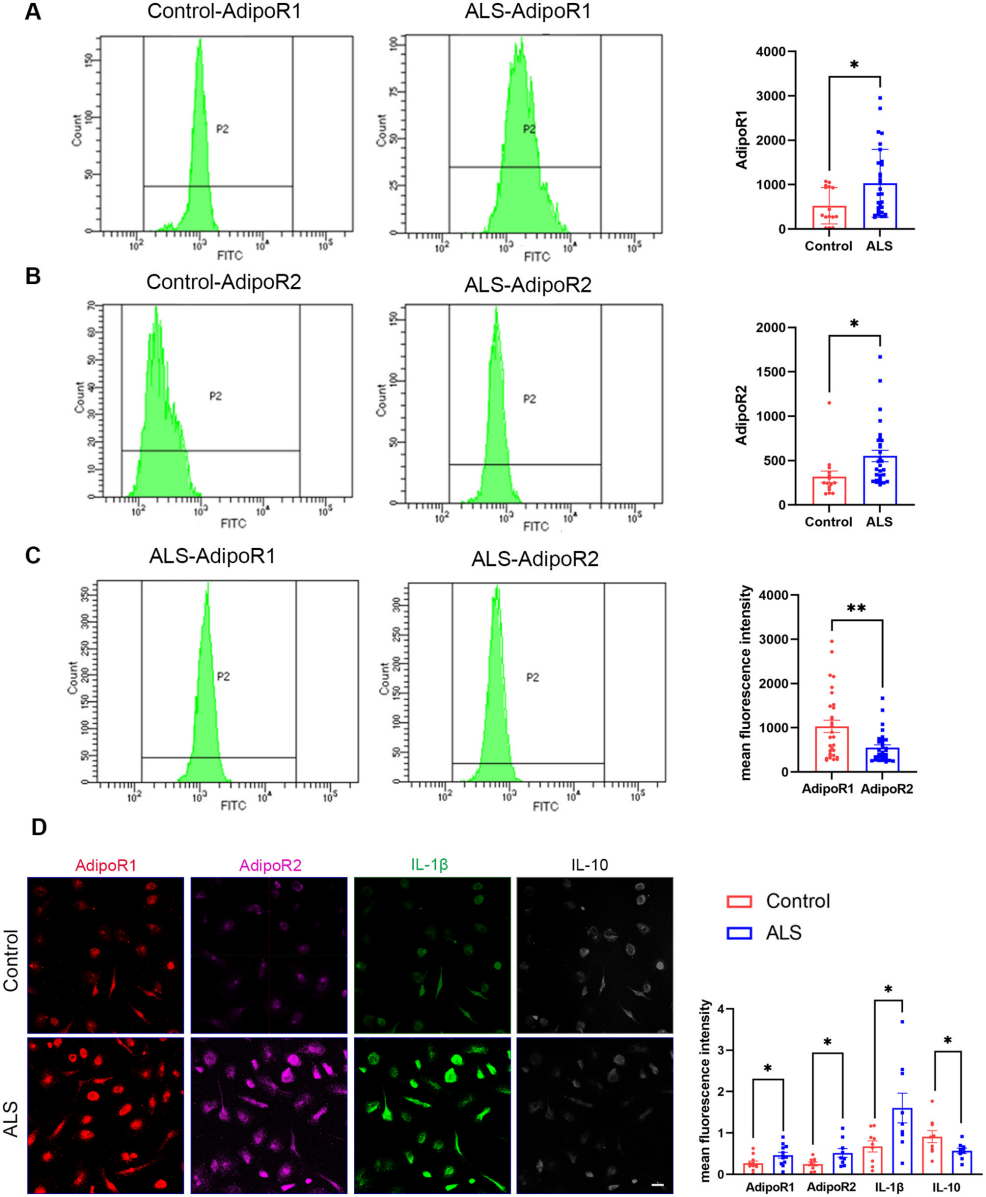
The levels of AdipoR1, AdipoR2, IL1β, and IL-10 in monocytes and monocyte-derived macrophages isolated from the blood of patients with ALS. **(A–C)** Results of flow cytometry for the expression levels of AdipoR1 and AdipoR2 on monocytes obtained from controls and ALS patients. Data represent the mean ± SEM. Statistical analysis was performed with an unpaired *t*-test, *P < 0.05; **P < 0.01. **(D)** Immunocytochemistry labeling for the expression of AdipoR1, AdipoR2, IL-1β, and IL-10 on macrophages from controls and patients with ALS (Scale bar= 20 μm). Data represent the mean ± SEM, *n* = 10 per group. Statistical analysis was performed with an unpaired *t*-test, *P < 0.05. ALS, amyotrophic lateral sclerosis; AdipoR1, Adiponectin receptor 1; AdipoR2, Adiponectin receptor 2.

Classical monocytes (CD14^++^CD16^−^) play a major role in phagocytosis and immunological responses (45), and prior experimental investigations have demonstrated that this monocytes could exacerbate disease progression in ALS (46). In addition, the main mechanism of action of adiponectin is mediated by AdipoR1 and AdipoR2 (27). Therefore, our main objective was to assess the expression levels of AdipoR1 and AdipoR2 in classical monocytes. Out of the 82 patients and 25 control participants, a total of 30 ALS patients and 15 control subjects were examined. Flow cytometry results indicated that the expression levels of AdipoR1 and AdipoR2 on monocytes were higher in ALS patients compared to the controls. Specifically, the expression level of AdipoR1 was 919.50 (1,038.5) in ALS patients and 444 (928) in controls, and the expression level of AdipoR2 was 512.50 (389) in ALS patients and 233 (195) in controls (Figures 2A, B). Simultaneously, the plasma adiponectin levels in ALS patients were shown to be lower than those in the control group (46.26 ± 12.85 vs. 63.05 ± 13.60, *P* = 0.002). In addition, it was observed that the expression level of AdipoR1 on monocytes was greater than that of AdipoR2 in ALS patients [919.50 (1,038.5) vs. 512.50 (389), *P* = 0.023] (Figure 2C).

Afterwards, we effectively stimulated the transformation of monocytes into macrophages *in vitro*, as established in our laboratory (47), and utilized immunocytochemistry (ICC) to assess the presence of AdipoR1 and AdipoR2 on these macrophages. Our findings revealed that the expresstion levels of AdipoR1 and AdipoR2 on macrophages from ALS patients were elevated compared to the controls in the absence of intervention (Figure 2D).

### 3.7 AdipoRon facilitated the polarization of macrophages from the M1 to the M2

**Figure 3:**
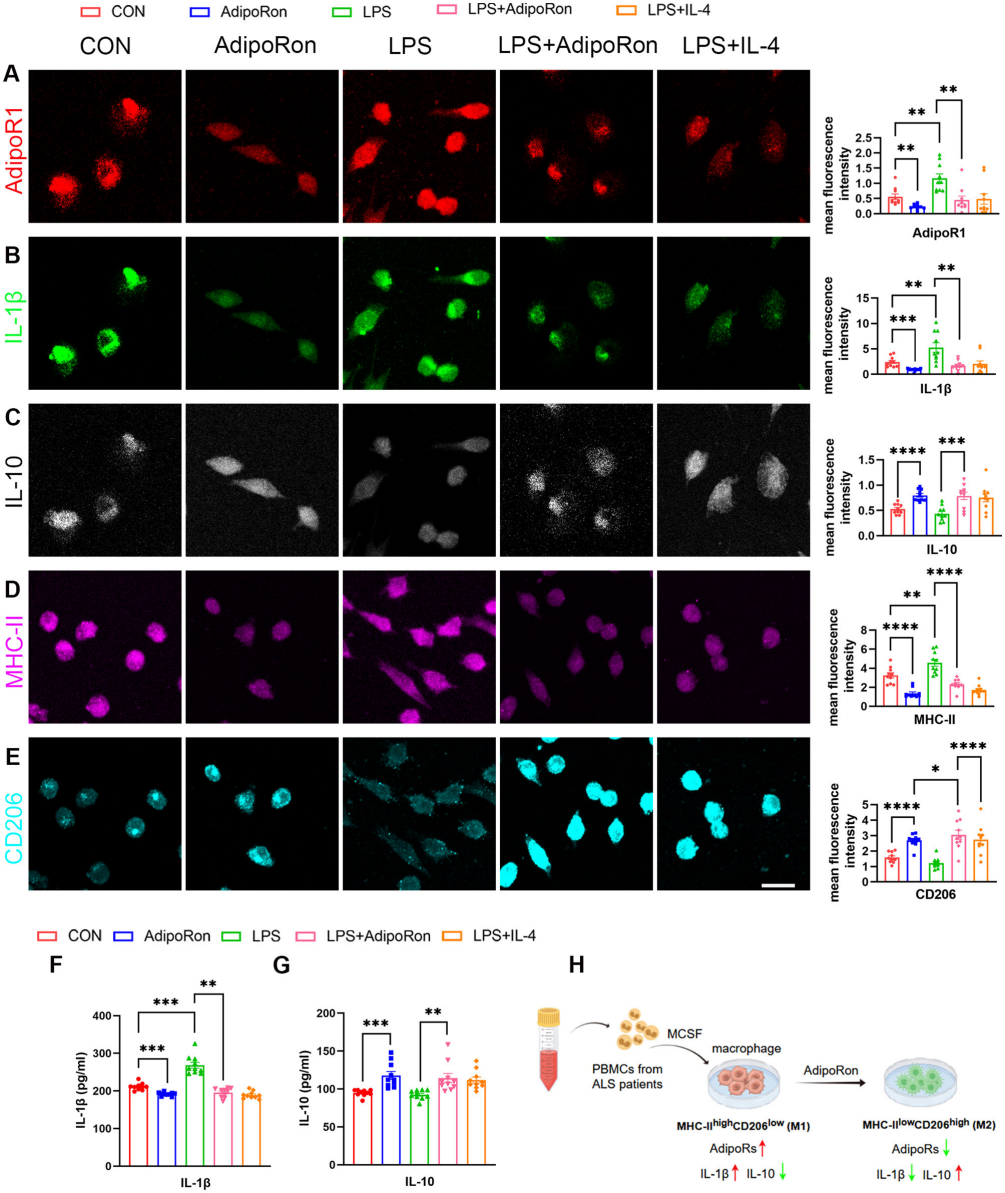
Inflammation and polarization of macrophages induced by AdipoRon. **(A–E)** Immunocytochemistry labeling and quantitative analysis of the fluorescence intensity for the expression of AdipoR1, IL-1β, IL-10, MHC-II, and CD206 on macrophages from patients with ALS, respectively (Scale bar = 20 μm). **(F, G)** Levels of IL-1β and IL-10 in the cell culture supernatants from patients with ALS under different interventions were determined by ELISA. **(H)** Schematic diagram of experimental design for investigate the impact of AdipoRon on macrophages polarization. Data represent the mean ± SEM, *n* = 10 per group. Statistical analysis was performed with one-way ANOVA, *P < 0.05; **P < 0.01; ***P < 0.001; ****P < 0.0001. AdipoR1, Adiponectin receptor 1; AdipoRon, Adiponectin receptor agonists; PBMC, peripheral blood mononuclear cell; ALS, amyotrophic lateral sclerosis; M-CSF, macrophage colony-stimulating factor.

To investigate the impact of increased AdipoRs on macrophages inflammation and polarization, we initially measured the expression levels of the prototypical proinfammatory cytokine IL-1β associated with M1 macrophages, and the anti-infammatory cytokine IL-10 associated with M2 macrophages derived from ALS patients. ICC results demonstrated that macrophages from ALS patients exhibited stronger expression of IL-1β, whereas showing reduced immunoreactivity for IL-10 compared to healthy controls (Figure 2D). Subsequently, we introduced the adiponectin analog AdipoRon into the culture medium of macrophages derived from ALS patients. ICC analysis revealed a significant reduction in the expression of IL-1β (Figure 3B) and MHC-II (Figure 3D) in AdipoRon-treated macrophages compared to untreated controls. Conversely, there was a notable increase in the expression of IL-10 (Figure 3C) and the CD206 (Figure 3E) in the AdipoRon-treated group. Notably, this characteristic phenotypic changes correlated with diminished AdipoR1 expression (Figure 3A). And these observations were corroborated by ELISA measurements of IL-1β (Figure 3F) and IL-10 levels (Figure 3G). Based on these findings, we proposed that AdipoRon may facilitate the polarization of macrophages from ALS patients from a pro-inflammatory M1 phenotype to an anti-inflammatory M2 phenotype (Figure 3H).

Next, to elucidate the impact of AdipoRon on macrophages polarization, we established models for M1 and M2-repolarization macrophages. The results of our study indicated that the group stimulated with LPS displayed a significant upregulation in the expression levels of IL-1β (Figures 3B, F) and MHC-II (Figure 3D) in comparison to the untreated group. Notably, this upregulation observed was reversed following the administration of AdipoRon, as evidenced by a drop in IL-1β (Figures 3B, F), and MHC-II (Figure 3D) levels, alongside with an increase in IL-10 (Figures 3C, G) and CD206 (Figures 3E) expression, mimicing the outcomes observed in the repolarization group. Consequently, these findings indicated that AdipoRon facilitated the polarization of macrophages from the M1 to the M2, thereby alleviating inflammation (Figure 3H).

### 3.8 AdipoRon-mediated polarization of M2 macrophages promoted neuronal proliferation and decreased apoptosis

**Figure 4:**
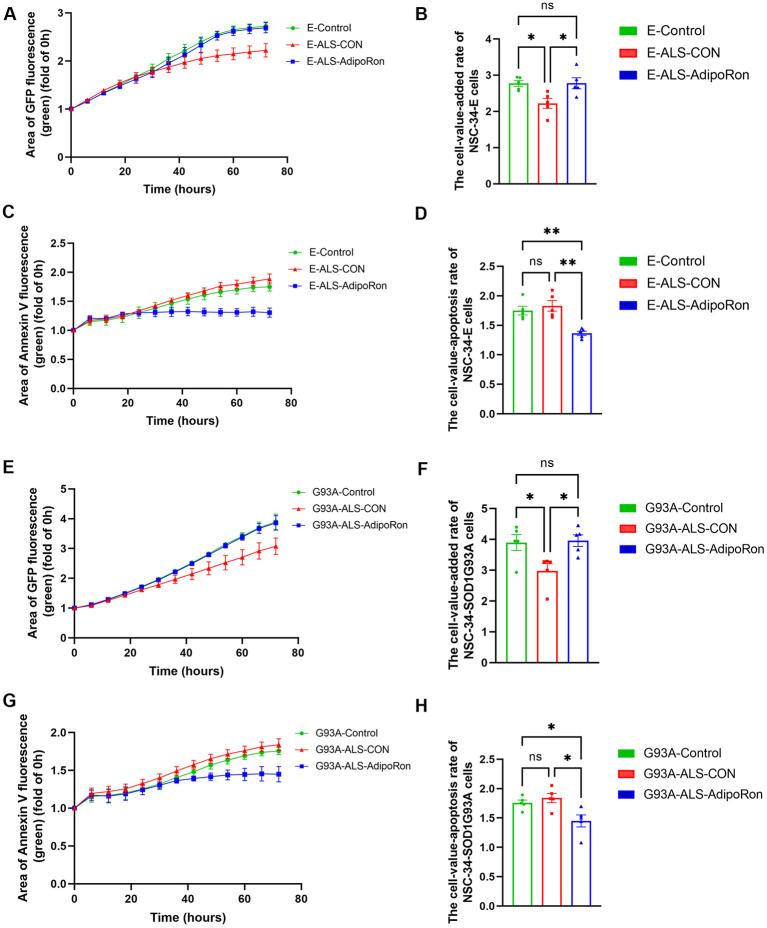
AdipoRon-mediated polarization of M2 macrophages affects neuronal proliferation and apoptosis. **(A, B)** Cell proliferation curve and the cell-value-added rate of NSC34-E cells under different treatments. **(C, D)** Cell apoptosis curve and the cell-value-apoptosis rate of NSC34-E cells under different treatments. **(E, F)** Cell proliferation curve and the cell-value-added rate of NSC34-hSOD1^G93A^ cells under different treatments. **(G, H)** Cell apoptosis curve and the cell-value-apoptosis rate of NSC34-hSOD1^G93A^ cells under different treatments. Data represent the mean ± SEM, *n* = 5 per group. Statistical analysis was performed with one-way ANOVA, *P < 0.05; **P < 0.01. AdipoRon, Adiponectin receptor agonists; AdipoR1, Adiponectin receptor 1; E, NSC34-E cell; G93A, NSC34-hSOD1^G93A^ cells.

Chronic systemic inflammation is implicated in the deterioration of MNs, resulting in axonal injuring and malfunction of neuromuscular junction. Given that AdipoRon has been shown to mitigate the pro-inflammatory response in activated macrophages, we extended our investigation by exposing NSC34-E and NSC34-hSOD1^G93A^ cells to the supernatant from macrophages of healthy controls and ALS patients, and examined their impacts on neuronal proliferation and apoptosis using live cell imaging technology. Our results indicated that the supernatant collected from macrophages obtained from individuals with ALS treated with AdipoRon had a signifcant effect on promoting neuronal proliferation (Figures 4A, B, E, F), and reducing apoptosis (Figures 4C, D, G, H) in comparison to untreated group of the ALS macrophages, which similar to the impact of the supernatant collected from healthy controls macrophages on the proliferation and apoptosis of NSC34-E (Figures 4A–D) and NSC34-hSOD1^G93A^ cells (Figures 4E–H). Collectively, our study suggested that the administration of AdipoRon may suppress pro-inflammatory macrophages derived from ALS patients and contribute to the protection of motor neurones.

## 4 Discussion

This study primarily aimed to explore the correlation between adiponectin and inflammatory mediators in ALS, and to investigate their associations with disease severity and progression, and to study the impact of adiponectin on peripheral blood monocytes and macrophages derived from ALS patients.

In the present study, ALS patients and control subjects were matched for sex and age. While there was a discrepancy in BMI between the two groups, further correlation analysis indicated no association between BMI and adiponectin or inflammatory mediators (Supplementary Table 3). Therefore, BMI did not influence the comparative outcomes between ALS patients and controls. To date, there has been no definitive consensus on adiponectin level between ALS patients and healthy controls. The majority of findings indicated a higher concentration of adiponectin in peripheral blood of ALS patients (31–33). But Bossolasco et al. (34) did not observe a notable disparity in adiponectin level between ALS patients and controls in either blood or cerebrospinal fluid. Contrarily, our study identified a lower concentration of adiponectin in ALS patients compared to controls. Bossolasco et al. (34) proposed that elevated adiponectin level could potentially function as a compensatory mechanism to counteract the gradual decline of neurones. Nonetheless, reduction in adipose tissue have been documented in ALS (48–50). Therefore, we hypothesized that adiponectin level in ALS might sustained or even elevated during the initial phases as a compensatory mechanism to mitigate neuronal damage. However, as the disease advances, a gradual reduction in adipose tissue could lead to a subsequent decline in adiponectin level. Additionally, our observations indicated that the mean ALSFRS-R in our patients cohort was lower than that in previous studies. Therefore, we speculated that the observed discrepancies may be attribute to the inclusion of patients at varying stages of illness. Moreover, patients with differing racial and sex compositions may also potentially influenced the outcomes. Therefore, extensive multi-center research is required to validate the adiponectin level in the peripheral blood of ALS patients.

Our study found that male ALS patients had significantly lower plasma adiponectin levels than females, which may be clinically relevant due to its association with higher δFS. Despite previous suggestions of estrogen's neuroprotective role in ALS (51), our additional stratified analysis showed no significant difference in adiponectin levels between premenopausal (n=8) and postmenopausal (n=23) women (57.86 ± 16.66 vs. 54.62 ± 12.52, *P* = 0.568), indicating that estrogen levels alone likely doesn't explain the sex-based disparity. There is a widely accepted understanding that ALS patients with bulbar onset exhibit a poorer prognosis. Nevertheless, no significant difference in adiponectin level was found between patients with bulbar onset and those with limb onset. We proposed that the sites of disease onset may not influence adiponectin secretion. Alternatively, the limited sample size might have hindered to accurately detect the disparities.

Numerous prior studies have examined the relationship between systemic inflammatory response and ALS (17, 52). In this investigation, we observed that the levels of IL-1β, IL-2, IL-6, IL-8, and TNF-α, were elevated, and the level of IL-10 was decreased in ALS patients compared to controls. Prior investigations (17, 53, 54), including our own study, have consistently demonstrated elevated levels of proinflammatory cytokines in the peripheral blood of ALS patients. Nevertheless, the findings regarding anti-inflammatory cytokines, such as IL-4, IL-10, and IL-13, in ALS patients have shown greater inconsistency in various research. Several investigations have documented markedly higher levels of IL-4, IL-10, and IL-13 in individuals with ALS (55–57), other studies have also reported negative results (58, 59). We hypothesized that the divergent outcomes regarding anti-inflammatory cytokines levels in ALS patients can be explained by the sensitivity of different detection methodologies, and the heterogeneity of the enrolled populations. Additionally, it is worth mentioning that there is currently limited documentation regarding chemokines in ALS patients. Trolese et al. (60) discovered that when CXCL13 was neutralized in fast-progressing ALS mice, it led to increased MNs impairment and skeletal muscles atrophy. In our investigation, no signicant disparities were detected in the concentrations of chemokines, including CX3CL1, CXCL10, CXCL13, and CCL2, between individuals with ALS and controls. Consequently, it is imperative to conduct larger studies to thoroughly investigate the levels of chemokines in ALS patients.

A persistent pro-inflammatory state could ultimately affect the severity and progression of the disease. This viewpoint has been corroborated by prior research (17, 52, 55) as well as the outcomes of our own study. Our investigation revealed that the concentrations of IL-1β, IL-2, IL-6, IL-8, and TNF-α were favorably linked to δFS, while being negatively correlated with ALSFRS-R in ALS patients. Besides, in our study, we observed higher concentrations of IL-2, IL-6, IL-8, TNF-α, and CXCL10, as well as lower level of IL-10 in the plasma of individuals with bulbar onset. Based on the association between IL-2, IL-6, IL-8, TNF-α and the severity and progression of the disease, we hypothesized that the pro-inflammatory response could potentially influence the prognosis of patients with varying onset sites. This underscores the crucial necessity of managing inflammation.

Extensive research has demonstrated that adiponectin exhibits anti-inflammatory properties (27). Adiponectin has been shown to suppress the activation of M1 macrophages and reduce the release of pro-inflammatory cytokines such as IFN-γ, IL-6, and TNF-α. Concurrently, adiponectin could stimulate M2 macrophages activation and promote the production of the anti-inflammatory cytokine IL-10 (61, 62). Furthermore, a lack of adiponectin can facilitate inflammation in the CNS (25, 63). Supplementation with adiponectin or AdipoRon may help manage the polarization and function of microglia, potentially ameliorating excessive inflammation. However, the relationship between adiponectin and inflammation in ALS remains unexplored. In the present study, we observed a strong negative correlation between plasma adiponectin level and IL-1β, IL-2, IL-6, IL-8, and TNF-α levels in ALS patients. Conversely, there was a positive correlation between adiponectin level and IL-4, IL-10 levels in ALS patients. Additionally, our findings indicated that adiponectin level had a substantial impact on the disease severity and progression. Specifically, there is a correlation between lower plasma adiponectin level and the reduced ALSFRS-R, as well as the elevated δFS in patients with ALS. Furthermore, a simple mediation study revealed that the impact of adiponectin on δFS was solely mediated by IL-2, or TNF-α, or IL-10. Thus, the regulatory role of adiponectin in ALS-related inflammation warrants further investigation.

Monocytes and macrophages play a crucial role in the natural defense system to protect the host from harm (64, 65). An increasing studies have demonstrated that peripheral blood monocytes and induced macrophages obtained from individuals with ALS exhibited noticeable proinflammatory traits (42). Additionally, preliminary immunohistochemical staining has identified the presence of macrophages infiltrating the spinal cord in patients with ALS (66). Furthermore, prior studies have shown that the progression of ALS is characterized by significant generation of proinflammatory cytokines, and increased activation and invasion of macrophages into peripheral nerves (67). Consequently, regulating macrophages activation could be a potentially effective therapeutic approach for decelerating the advancement of ALS. To elucidate the role of adiponectin in modulating inflammation in individuals with ALS, we conducted a study utilizing peripheral blood monocytes and induced macrophages. Our findings indicated an upregulation of AdipoR1 and AdipoR2 expression in peripheral blood monocytes and macrophages from ALS patients, and this upregulation is accompanied by an elevation in the release of proinflammatory cytokines in macrophages. We proposed that the increase in AdipoR1 and AdipoR2 expression served as a compensatory mechanism in response to the diminished plasma adiponectin level in ALS patients. Moreover, administration of AdipoRon resulted in the downregulation of AdipoR1 expression, a reduction in proinflammatory cytokine levels, and the regulation of macrophages shifting toward the M2 phenotype. Concurrently, AdipoRon demonstrated the potential to diminish the detrimental effects of proinflammatory macrophages on neurons.

However, our study was subject to many constraints. Firstly, the cross-sectional design precluded longitudinal assessment of disease progression in patients with ALS, thereby restricting our ability to comprehensively evaluate the lasting impact of adiponectin on the patient population. Additionally, our research was conducted as a single-center study with a relatively homogenous population and a limited sample. Furthermore, we did not investigate the specific mechanisms by which adiponectin regulates macrophage inflammation.

Our study found a significant link between adiponectin and inflammation in ALS patients, offering insights into neuroinflammation regulation and suggesting new ALS treatments. Future research should explore adiponectin's role in ALS models to confirm its anti-inflammatory and neuroprotective effects, potentially using adiponectin mimetics or pathway modulators. Clinically, tracking adiponectin levels over time could establish it as a biomarker for inflammation and treatment response. Additionally, combining adiponectin with other markers could improve prognosis and aid in personalized therapy approaches.

## Data Availability

The original contributions presented in the study are included in the article/Supplementary material, further inquiries can be directed to the corresponding authors.

## References

[1] Feldman EL Goutman SA Petri S Mazzini L Savelieff MG Shaw PJ. Amyotrophic lateral sclerosis. Lancet. (2022) 400:1363–80. 10.1016/S0140-6736(22)01272-7 36116464 PMC10089700

[2] Xu L Liu T Liu L Yao X Chen L Fan D. (2020). Global variation in prevalence and incidence of amyotrophic lateral sclerosis: a systematic review and meta-analysis. J Neurol. 267:944–53. 10.1007/s00415-019-09652-y 31797084

[3] Saitoh Y Takahashi Y. Riluzole for the treatment of amyotrophic lateral sclerosis. Neurodegener Dis Manag. (2020) 10:343–55. 10.2217/nmt-2020-0033 32847483

[4] Witzel S Maier A Steinbach R Grosskreutz J Koch JC Sarikidi A. Safety and effectiveness of long-term intravenous administration of edaravone for treatment of patients with amyotrophic lateral sclerosis. JAMA Neurol. 79:121–30. 10.1001/jamaneurol.2021.4893 35006266 PMC8749709

[5] Masrori P Beckers J Gossye H Van Damme P. The role of inflammation in neurodegeneration: novel insights into the role of the immune system in C9orf72 HRE-mediated ALS/FTD. Mol Neurodegener. (2022) 17:22. 10.1186/s13024-022-00525-z 35303907 PMC8932121

[6] Murdock BJ Zhou T Kashlan SR Little RJ Goutman SA Feldman EL. Correlation of peripheral immunity with rapid amyotrophic lateral sclerosis progression. JAMA Neurol. 74:1446–54. 10.1001/jamaneurol.2017.2255 28973548 PMC5822195

[7] Jiang Q Wei Q Zhang L Yang T Lin J Xiao Y. Peripheral immunity relate to disease progression and prognosis in amyotrophic lateral sclerosis. Amyotroph Lateral Scler Frontotemporal Degener. (2020) 25:465–74. 10.1080/21678421.2024.2306969 38270154

[8] Piccoli T Castro F La Bella V Meraviglia S Di Simone M Salemi G. Role of the immune system in amyotrophic lateral sclerosis. Analysis of the natural killer cells and other circulating lymphocytes in a cohort of ALS patients. BMC Neurol. (2023) 23:222. 10.1186/s12883-023-03255-x 37296379 PMC10251617

[9] Nona RJ Henderson RD McCombe PA. Neutrophil-to-lymphocyte ratio at diagnosis as a biomarker for survival of amyotrophic lateral sclerosis. Amyotroph Lateral Scler Frontotemporal Degener. (2024) 25:452–64. 10.1080/21678421.2024.2351187 38745425

[10] Cotet C Alarcan H Hérault O Corcia P Vourc'h P Andres CR. Neutrophil to lymphocyte ratio as a prognostic marker in amyotrophic lateral sclerosis. Biomolecules. (2023) 13:1689. 10.3390/biom13121689 38136561 PMC10741910

[11] Chiu IM Phatnani H Kuligowski M Tapia JC Carrasco MA Zhang M. Activation of innate and humoral immunity in the peripheral nervous system of ALS transgenic mice. Proc Natl Acad Sci U S A. (2009) 106:20960–5. 10.1073/pnas.0911405106 19933335 PMC2791631

[12] Angelini DF De Angelis F Vacca V Piras E Parisi C Nutini M. Very early involvement of innate immunity in peripheral nerve degeneration in SOD1-G93A mice. Front Immunol. (2020) 11:575792. 10.3389/fimmu.2020.575792 33329541 PMC7714949

[13] Trias E Kovacs M King PH Si Y Kwon Y Varela V. Schwann cells orchestrate peripheral nerve inflammation through the expression of CSF1, IL-34, and SCF in amyotrophic lateral sclerosis. Glia. (2020) 68:1165–81. 10.1002/glia.23768 31859421 PMC7269115

[14] Kawamata T Akiyama H Yamada T McGeer PL. Immunologic reactions in amyotrophic lateral sclerosis brain and spinal cord tissue. Am J Pathol. (1992) 140:691–707. 1347673 PMC1886170

[15] Tondo G Iaccarino L Cerami C Vanoli GE Presotto L Masiello V. 11C-PK11195 PET-based molecular study of microglia activation in SOD1 amyotrophic lateral sclerosis. Ann Clin Transl Neurol. (2020) 7:1513–23. 10.1002/acn3.51112 32762033 PMC7480909

[16] Staats KA Borchelt DR Tansey MG Wymer J. Blood-based biomarkers of inflammation in amyotrophic lateral sclerosis. Mol Neurodegeneration. (2022) 17:11. 10.1186/s13024-022-00515-1 35073950 PMC8785449

[17] Sun Q Huo Y Bai J Wang H Wang H Yang F. Inflammatory cytokine levels in patients with sporadic amyotrophic lateral sclerosis. Neurodegener Dis. (2021) 21:87–92. 10.1159/000522078 35124669

[18] Appel SH Beers DR Zhao W. Amyotrophic lateral sclerosis is a systemic disease: peripheral contributions to inflammation-mediated neurodegeneration. Curr Opin Neurol. (2021) 34:765–72. 10.1097/WCO.0000000000000983 34402459

[19] Mora JS Genge A Chio A Estol CJ Chaverri D Hernández M. Masitinib as an add-on therapy to riluzole in patients with amyotrophic lateral sclerosis: a randomized clinical trial. Amyotroph Lateral Scler Frontotemporal Degener. (2020) 21:5–14. 10.1080/21678421.2019.1632346 31280619

[20] Mora JS Bradley WG Chaverri D Hernández-Barral M Mascias J Gamez J. Long-term survival analysis of masitinib in amyotrophic lateral sclerosis. Ther Adv Neurol Disord. (2021) 14:17562864211030365. 10.1177/17562864211030365 34457038 PMC8388186

[21] Zhang R Azhir A McGrath MS. Respiratory function improvement and lifespan extension following immunotherapy with NP001 support the concept that amyotrophic lateral sclerosis (ALS) is an immuno-neurologic disease. Int J Mol Sci. (2025) 26:4349. 10.3390/ijms26094349 40362586 PMC12072840

[22] Forrest BD Goyal NA Fleming TR Bracci PM Brett NR Khan Z. The effectiveness of NP001 on the long-term survival of patients with amyotrophic lateral sclerosis. Biomedicines. (2024) 12:2367. 10.3390/biomedicines12102367 39457678 PMC11504292

[23] Thonhoff JR Beers DR Zhao W Pleitez M Simpson EP Berry JD. Expanded autologous regulatory T-lymphocyte infusions in ALS: a phase I, first-in-human study. Neurol Neuroimmunol Neuroinflamm. (2018) 5:e465. 10.1212/NXI.0000000000000465 29845093 PMC5961523

[24] Camu W Mickunas M Veyrune JL Payan C Garlanda C Locati M. Repeated 5-day cycles of low dose aldesleukin in amyotrophic lateral sclerosis (IMODALS): a phase 2a randomised, double-blind, placebo-controlled trial. EBioMedicine. (2020) 59:102844. 10.1016/j.ebiom.2020.102844 32651161 PMC7502670

[25] Rijnsburger M Djuric N Mulder IA de Vries HE. Adipokines as immune cell modulators in multiple sclerosis. Int J Mol Sci. (2021) 22:10845. 10.3390/ijms221910845 34639186 PMC8509121

[26] Liu H Wu X Luo J Zhao L Li X Guo H. Adiponectin peptide alleviates oxidative stress and NLRP3 inflammasome activation after cerebral ischemia-reperfusion injury by regulating AMPK/GSK-3β. Exp Neurol. (2020) 329:113302. 10.1016/j.expneurol.2020.113302 32275928

[27] Thundyil J Pavlovski D Sobey CG Arumugam TV. Adiponectin receptor signalling in the brain. Br J Pharmacol. (2021) 165:313–27. 10.1111/j.1476-5381.2011.01560.x 21718299 PMC3268187

[28] Ng RCL Chan KH. Potential neuroprotective effects of adiponectin in Alzheimer's disease. Int J Mol Sci. (2017) 18:592. 10.3390/ijms18030592 28282917 PMC5372608

[29] He K Nie L Ali T Wang S Chen X. Adiponectin alleviated Alzheimer-like pathologies via autophagy-lysosomal activation. Aging Cell. (2021) 20:e13514. 10.1111/acel.13514 34775673 PMC8672778

[30] Ng RCL Jian M Ma OKF Bunting M Kwan JSC Zhou GJ. Chronic oral administration of adipoRon reverses cognitive impairments and ameliorates neuropathology in an Alzheimer's disease mouse model. Mol Psychiatry. (2021) 26:5669–89. 10.1038/s41380-020-0701-0 32132650

[31] Nagel G Peter RS Rosenbohm A Koenig W Dupuis L Rothenbacher D. Adipokines, C-reactive protein and amyotrophic lateral sclerosis - results from a population- based ALS registry in Germany. Sci Rep. (2017) 7:4374. 10.1038/s41598-017-04706-5 28663573 PMC5491500

[32] Li JY Cui LY Sun XH Shen DC Yang XZ Liu Q. Alterations in metabolic biomarkers and their potential role in amyotrophic lateral sclerosis. Ann Clin Transl Neurol. (2022) 9:1027–38. 10.1002/acn3.51580 35584112 PMC9268864

[33] Ngo ST Steyn FJ Huang L Mantovani S Pfluger CMM Woodruff TM. Altered expression of metabolic proteins and adipokines in patients with amyotrophic lateral sclerosis. J Neurol Sci. (2015) 357:22–7. 10.1016/j.jns.2015.06.053 26198021

[34] Bossolasco P Cancello R Doretti A Morelli C Silani V Cova L. Adiponectin levels in the serum and cerebrospinal fluid of amyotrophic lateral sclerosis patients: possible influence on neuroinflammation? J Neuroinflammation. (2017) 14:85. 10.1186/s12974-017-0861-2 28427413 PMC5397697

[35] Brooks BR Miller RG Swash M Munsat TL. El Escorial revisited: revised criteria for the diagnosis of amyotrophic lateral sclerosis. Amyotroph Lateral Scler Other Motor Neuron Disord. (2000) 1:293–9. 10.1080/146608200300079536 11464847

[36] Von Elm E Altman DG Egger M Pocock SJ Gøtzsche PC Vandenbroucke JP. The strengthening the reporting of observational studies in epidemiology (STROBE) statement: guidelines for reporting observational studies. J Clin Epidemiol. (2014) 12:1495–9. 10.1016/j.ijsu.2014.07.013 18313558

[37] Yeudall S Upchurch CM Seegren PV Pavelec CM Greulich J Lemke MC. Macrophage acetyl-CoA carboxylase regulates acute inflammation through control of glucose and lipid metabolism. Sci Adv. (2022) 8:eabq1984. 10.1126/sciadv.abq1984 36417534 PMC9683712

[38] Cashman NR Durham HD Blusztajn JK Oda K Tabira T Shaw IT. Neuroblastoma x spinal cord (NSC) hybrid cell lines resemble developing motor neurons. Dev Dyn. (1992) 194:209–21. 10.1002/aja.1001940306 1467557

[39] Liu Y Duan W Guo Y Li Z Han H Zhang S. A new cellular model of pathological TDP-43: the neurotoxicity of stably expressed CTF25 of TDP-43 depends on the proteasome. Neuroscience. (2014) 281:88–98. 10.1016/j.neuroscience.2014.09.043 25270903

[40] Bai L Wang Y Huo J Li S Wen Y Liu Q. Simvastatin accelerated motoneurons death in SOD1G93A mice through inhibiting Rab7-mediated maturation of late autophagic vacuoles. Cell Death Dis. (2021) 12:392. 10.1038/s41419-021-03669-w 33846297 PMC8041862

[41] Qi W Yan L Liu Y Zhou X Li R Wang Y. Simvastatin aggravates impaired autophagic flux in NSC34-hSOD1G93A cells through inhibition of geranylgeranyl pyrophosphate synthesis. Neuroscience. (2019) 409:130–41. 10.1016/j.neuroscience.2019.04.034 31051215

[42] Du Y Zhao W Thonhoff JR Wang J Wen S Appel SH. Increased activation ability of monocytes from ALS patients. Exp Neurol. (2020) 328:113259. 10.1016/j.expneurol.2020.113259 32105709

[43] Jung S Schwartz M. Non-identical twins - microglia and monocyte-derived macrophages in acute injury and autoimmune inflammation. Front Immunol. (2012) 3:89. 10.3389/fimmu.2012.00089 22566968 PMC3345364

[44] Chiot A Zaïdi S Iltis C Ribon M Berriat F Schiaffino L. Modifying macrophages at the periphery has the capacity to change microglial reactivity and to extend ALS survival. Nat Neurosci. (2020) 23:1339–51. 10.1038/s41593-020-00718-z 33077946

[45] Kapellos TS Bonaguro L Gemünd I Reusch N Saglam A Hinkley ER. Human monocyte subsets and phenotypes in major chronic inflammatory diseases. Front Immunol. (2019) 10:2035. 10.3389/fimmu.2019.02035 31543877 PMC6728754

[46] Zondler L Feiler MS Freischmidt A Ruf WP Ludolph AC Danzer KM. Impaired activation of ALS monocytes by exosomes. Immunol Cell Biol. (2017) 95:207–14. 10.1038/icb.2016.89 27616750

[47] Yang J Xin C Huo J Li X Dong H Liu Q. Rab geranylgeranyltransferase subunit beta as a potential indicator to assess the progression of amyotrophic lateral sclerosis. Brain Sci. (2023) 13:1531. 10.3390/brainsci13111531 38002490 PMC10670085

[48] Lindauer E Dupuis L Müller HP Neumann H Ludolph AC Kassubek J. Adipose tissue distribution predicts survival in amyotrophic lateral sclerosis. PLoS ONE. (2013) 8:e67783. 10.1371/journal.pone.0067783 23826340 PMC3694869

[49] Vernikouskaya I Müller HP Felbel D Roselli F Ludolph AC Kassubek J. Body fat compartment determination by encoder–decoder convolutional neural network: application to amyotrophic lateral sclerosis. Sci Rep. (2022) 12:5513. 10.1038/s41598-022-09518-w 35365743 PMC8976026

[50] Dupuis L Oudart H René F de Aguilar JLG Loeffler JP. Evidence for defective energy homeostasis in amyotrophic lateral sclerosis: benefit of a high-energy diet in a transgenic mouse model. Proc Natl Acad Sci U S A. (2004) 101:11159–64. 10.1073/pnas.0402026101 15263088 PMC503756

[51] Yan L Liu Y Sun C Zheng Q Hao P Zhai J. Effects of ovariectomy in an hSOD1-G93A transgenic mouse model of amyotrophic lateral sclerosis (ALS). Med Sci Monit. (2018) 24:678–86. 10.12659/MSM.908786 29394243 PMC5806477

[52] Olesen MN Wuolikainen A Nilsson AC Wirenfeldt M Forsberg K Madsen JS. Inflammatory profiles relate to survival in subtypes of amyotrophic lateral sclerosis. Neurol Neuroimmunol Neuroinflamm. (2020) 7:e697. 10.1212/NXI.0000000000000697 32123048 PMC7136052

[53] Tortelli R Zecca C Piccininni M Benmahamed S Dell'Abate MT Barulli MR. Plasma Inflammatory Cytokines Are Elevated in ALS. Front Neurol. (2020) 11:552295. 10.3389/fneur.2020.552295 33281700 PMC7691268

[54] Xu CZ Huan X Luo SS Zhong HH Zhao CB Chen Y. Serum cytokines profile changes in amyotrophic lateral sclerosis Heliyon. (2024) 10:e28553. 10.1016/j.heliyon.2024.e28553 38596011 PMC11002056

[55] Lu CH Allen K Oei F Leoni E Kuhle J Tree T. Systemic inflammatory response and neuromuscular involvement in amyotrophic lateral sclerosis. Neurol Neuroimmunol Neuroinflamm. (2016) 3:e244. 10.1212/NXI.0000000000000244 27308305 PMC4897985

[56] Polverino A Rucco R Stillitano I Bonavita S Grimaldi M Minino R. In amyotrophic lateral sclerosis blood cytokines are altered, but do not correlate with changes in brain topology. Brain Connect. (2020) 10:411–21. 10.1089/brain.2020.0741 32731760

[57] Shi N Kawano Y Tateishi T Kikuchi H Osoegawa M Ohyagi Y. Increased IL-13-producing T cells in ALS: positive correlations with disease severity and progression rate. J Neuroimmunol. (2007) 182:232–5. 10.1016/j.jneuroim.2006.10.001 17097743

[58] Hu Y Cao C Qin XY Yu Y Yuan J Zhao Y. Increased peripheral blood inflammatory cytokine levels in amyotrophic lateral sclerosis: a meta-analysis study. Sci Rep. (2017) 7:9094. 10.1038/s41598-017-09097-1 28831083 PMC5567306

[59] Ehrhart J Smith AJ Kuzmin-Nichols N Zesiewicz TA Jahan I Shytle RD. Humoral factors in ALS patients during disease progression. J Neuroinflammation. (2015) 12:127. 10.1186/s12974-015-0350-4 26126965 PMC4487852

[60] Trolese MC Mariani A Terao M De Paola M Fabbrizio P Sironi F. CXCL13/CXCR5 signalling is pivotal to preserve motor neurons in amyotrophic lateral sclerosis. eBioMedicine. (2020) 62:103097. 10.1016/j.ebiom.2020.103097 33161233 PMC7670099

[61] Wolf AM Wolf D Rumpold H Enrich B Tilg H. Adiponectin induces the anti-inflammatory cytokines IL-10 and IL-1RA in human leukocytes. Biochem Biophys Res Commun. (2004) 323:630–5. 10.1016/j.bbrc.2004.08.145 15369797

[62] Luo Y Liu M. Adiponectin: a versatile player of innate immunity. J Mol Cell Biol. (2016) 8:120–8. 10.1093/jmcb/mjw012 26993045 PMC4816149

[63] He K Nie L Ali T Liu Z Li W Gao R. Adiponectin deficiency accelerates brain aging via mitochondria-associated neuroinflammation. Immun Ageing. (2023) 20:15. 10.1186/s12979-023-00339-7 37005686 PMC10067304

[64] Sica A Mantovani A. Macrophage plasticity and polarization: *in vivo* veritas. J Clin Invest. (2012) 122:787–95. 10.1172/JCI59643 22378047 PMC3287223

[65] Gordon S Martinez FO. Alternative activation of macrophages. Nat Rev Immunol. (2003) 3:23–35. 10.1038/nri978 12511873

[66] Troost D Van den Oord JJ Vianney de. Jong JM. Immunohistochemical characterization of the inflammatory infiltrate in amyotrophic lateral sclerosis. Neuropathol Appl Neurobiol. (1990) 16:401–10. 10.1111/j.1365-2990.1990.tb01276.x 2263315

[67] Trias E Ibarburu S Barreto-Núñez R Varela V Moura IC Dubreuil P. Evidence for mast cells contributing to neuromuscular pathology in an inherited model of ALS. JCI Insight. 2:e95934. 10.1172/jci.insight.95934 29046475 PMC5846907

