# Supplementary material for: Unlocking amyotrophic lateral sclerosis: the role of adiponectin in inflammation and disease progression
Source: Front Neurol. 2025 Jul 4;16:1605822. doi: 10.3389/fneur.2025.1605822 (PMC12270890; doi:10.3389/fneur.2025.1605822)
Supplement: Supplementary file 1 [file Table_1.docx]

**Supplementary Materials:**

Supplementary Table 1: An analysis of the correlation between plasma adiponectin, inflammatory mediators, and the ALSFRS-R, δFS in patients with ALS;

Supplementary Table 2: An analysis of the correlation between plasma adiponectin and inflammatory mediators in patients with ALS;

Supplementary Table 3: An analysis of the correlation between plasma adiponectin, inflammatory mediators and BMI in ALS patients.

**Supplementary Table 1**

An analysis of the correlation between plasma adiponectin, inflammatory mediators, and the ALSFRS-R, δFS in patients with ALS.

|  | ALSFRS-R | | δFS | |
| --- | --- | --- | --- | --- |
|  | *P*-Value | r | *P*-Value | r |
| ***Adipokine*** |  | |  | |
| Adiponectin (ng/ml) | ***P*= 0.001**** | r= 0.434 | ***P*< 0.001**** | r=-0.761 |
| ***Pro-inflammatory cytokines*** |  | |  | |
| IL-1β (pg/ml) | *P*= 0.080 | r=-0.234 | ***P*< 0.001**** | r= 0.639 |
| IL-2 (pg/ml) | ***P*= 0.001**** | r=-0.412 | ***P*< 0.001**** | r= 0.786 |
| IL-6 (pg/ml) | ***P*= 0.002**** | r=-0.399 | ***P*< 0.001**** | r= 0.674 |
| IL-8 (pg/ml) | ***P*= 0.002**** | r=-0.410 | ***P*< 0.001**** | r= 0.694 |
| TNF-α (pg/ml) | ***P*< 0.001**** | r=-0.510 | ***P*< 0.001**** | r= 0.738 |
| ***anti-inflammatory cytokines*** |  | |  | |
| IL-4 (pg/ml) | *P*= 0.258 | r= 0.152 | *P*= 0.078 | r=-0.235 |
| IL-10 (pg/ml) | ***P*< 0.001**** | r= 0.471 | ***P*< 0.001**** | r=-0.801 |
| IL-13 (pg/ml) | *P*= 0.358 | r=-0.124 | *P*= 0.621 | r= 0.067 |
| TGF-β (pg/ml) | *P*= 0.342 | r=-0.128 | *P*= 0.394 | r= 0.115 |
| ***Chemokines*** |  | |  | |
| CXCL13 (pg/ml) | *P*= 0.691 | r=-0.054 | *P*= 0.906 | r= 0.016 |
| CXCL10 (pg/ml) | *P*= 0.440 | r=-0.104 | *P*= 0.116 | r= 0.211 |
| CX3CL1 (ng/ml) | *P*= 0.462 | r= 0.099 | *P*= 0.849 | r=-0.026 |
| CCL2 (pg/ml) | *P*= 0.507 | r= 0.090 | *P*= 0.394 | r=-0.115 |

ALSFRS-R, revised ALS functional rating scale; δFS= 48-ALSFRS-R at the time of diagnosis/disease duration; *, *P* < 0.05; **, *P* < 0.01.

**Supplementary Table 2**

An analysis of the correlation between plasma adiponectin and inflammatory mediators in patients with ALS.

|  | adiponectin |  |
| --- | --- | --- |
|  | *P*-value | r |
| IL-1β | **<0.001**** | -0.674 |
| IL-2 | **<0.001**** | -0.676 |
| IL-6 | **<0.001**** | -0.564 |
| IL-8 | **<0.001**** | -0.566 |
| TNF-α | **<0.001**** | -0.682 |
| IL-4 | **0.015*** | 0.320 |
| IL-10 | **<0.001**** | 0.693 |
| IL-13 | 0.602 | -0.071 |
| TGFβ | 0.289 | -0.143 |
| CXCL13 | 0.311 | -0.136 |
| CXCL10 | 0.228 | -0.162 |
| CX3CL1 | 0.588 | 0.073 |
| CCL2 | 0.873 | 0.022 |

*, *P* < 0.05; **, *P* < 0.01.

**Supplementary Table 3**

An analysis of the correlation between plasma adiponectin, inflammatory mediators and BMI in ALS patients.

|  | BMI |  |
| --- | --- | --- |
|  | *P*-value | r |
| adiponectin | 0.310 | -0.137 |
| IL-1β | 0.100 | -0.220 |
| IL-2 | 0.197 | 0.173 |
| IL-6 | 0.290 | 0.290 |
| IL-8 | 0.149 | 0.194 |
| TNF-α | 0.928 | -0.120 |
| IL-4 | 0.838 | -0.280 |
| IL-10 | 0.764 | -0.410 |
| IL-13 | 0.269 | 0.149 |
| TGFβ | 0.526 | -0.086 |
| CXCL13 | 0.276 | -0.147 |
| CXCL10 | 0.716 | -0.049 |
| CX3CL1 | 0.790 | 0.036 |
| CCL2 | 0.494 | -0.092 |

BMI, body mass index.
